# Effect of the Physical Environment on the Health-related Quality of Life of the Low-income Korean Elderly Population

**Published:** 2018-12

**Authors:** Seonhye LEE, Chang Heon CHEONG

**Affiliations:** 1. Dept. of Nursing, Gyeongnam National University of Science and Technology, 33, Dongjin-ro, Jinju-si, Gyeongsangnam-do, 52725, Republic of Korea; 2. Dept. of Architectural Engineering, Gyeongnam National University of Science and Technology, 33, Dongjin-ro, Jinju-si, Gyeongsangnam-do, 52725, Republic of Korea

**Keywords:** Elderly, Health, Korean community health survey, Low income, Quality of life

## Abstract

**Background:**

The increasing number of elderly citizens due to changes in the social structure is of national interest. This study aimed to provide basic data for devising policies to promote the quality of life of elderly National Basic Livelihood Security System (NBLSS) beneficiaries in South Korea by identifying the effects of their general characteristics and physical environment on their health-related quality of life (HRQoL).

**Methods:**

Using the 2013 Korean Community Health Survey (KCHS) raw data, we analyzed 3537 NBLSS beneficiaries aged 65 yr and older. HRQoL was measured using the Euro QoL five-dimension questionnaire (EQ-5D). Hierarchical multiple regression analyses were used to predict the EQ-5D scores.

**Results:**

The explanatory power for HRQoL increased to 21.4% when the general characteristics and physical environment were included. HRQoL showed statistically significant differences in the environmental variable, level of safety (*P*=0.001), natural environment (*P*=0.001), living environment (*P*=0.001), traffic condition (*P*<0.001), and access to health services (*P*<0.001). Physical environment positively correlated with HRQoL(r=.119, *P*<0.001), thus confirming its influence (ß=.092, *P*<0.001).

**Conclusion:**

We should strive to manage the physical environment to improve the quality of life of elderly NBLSS beneficiaries.

## Introduction

The National Basic Livelihood Security System (NBLSS) was implemented as a part of a public assistance policy to guarantee sustainable living for people who find it difficult to survive and develop their self-supporting abilities in South Korea (1). As of 31 December 2014, the number of NBLSS beneficiaries reached 1.33 million (905000 households), accounting for 2.6% of the total population. The ratio of NBLSS beneficiaries to the total population per age group is the highest among the elderly aged 65 yr or older (2). The proportion of recipients is increasing because of the accelerated population aging, changes in family structure, and lack of financial reserve, which demand attention and adequate measures to improve the quality of life (QoL) of the low-income elderly population (1, 3).

A combination of various physical and mental factors, such as social retirement, changed roles within the family, and physical aging may under-mine the QoL of the elderly (4). Health-related quality of life (HRQoL) refers to one’s subjective well-being because of the impact of one’s health status on the purpose, expectations, standards, and interests of life (5). HRQoL is affected by personal environment, community environment, and public policy factors (6). HRQoL measured with the Euro QoL five-dimension questionnaire (EQ-5D) provides the key criteria for the assessment of public health policies, understanding of the disadvantaged classes, and evaluation of policy proposals and decision-making (7). The EQ-5D is currently used in HRQoL research on diverse social strata globally (8–12).

Rapid escalation of healthcare and medical costs due to the growing prevalence of obesity, asthma, heart disease, and cancer has heightened the need for research on urban environment and health (13), and previous studies assessed the QoL of urban residents in connection to their physical environment (14–16). The physical environment influences the individual’s health in homes, neighborhoods (e.g., environments that increase physical activity, such as boardwalk, bicycle path, etc.) and workplace, which affect the HRQoL (6). The physical environment exerts significant influence on the subjective health status of urban and rural residents (17), suggesting the importance of considering them when developing health promotion programs for local residents. The environment is especially critical for the elderly, as reduced mobility significantly shrinks their access to places in daily life, and the physical distance to major services and facilities has a direct effect on their QoL (18). Thus, understanding the association between HRQoL and physical environment among the low-income elderly population could contribute to the formulation of policies for local communities.

However, nearly all studies assessing the QoL of elderly Korean citizens have only focused on demographic features and health status at the individual level (3, 5, 19), and there has been no investigation of the physical environment at the level of local communities. This study aimed to examine the association between the HRQoL, general characteristics, and physical environment of the NBLSS recipients elderly in Korea. The ultimate goal was to contribute to creating healthier community environments and improve the HRQoL of the target population.

## Materials and Methods

### Study design and databases

This study used a descriptive and cross-sectional research design. The 2013 Korean Community Health Survey (KCHS) data were used for this study. The Korea Centers for Disease Prevention and Control (KCDC) conducts a community health survey annually by sending trained surveyors to investigate a representative sample (n=900) from each of the 254 cities, districts, and borough public health centers in Korea using a computer-assisted personal interviewing technique. The questionnaire consisted of general characteristics, diseased condition, the EQ-5D, environmental factors, etc. In 2013, 228781 people were surveyed, of which 3645 individuals were elderly (≥65 yr) NBLSS beneficiaries. After excluding 108 individuals for having omissions in the EQ-5D scores, data from 3537 individuals were included in the analysis.

### Ethical review

The raw dataset was obtained from the KCDC after registering to access the database. We submitted a research plan for the use of raw data was electronically submitted to the KCDC homepage and obtained an approval for using the data (No. 2013-06EXP-01-3C). All personal identification information was deleted from the data before analysis. Participants in this survey provided their informed consent.

### Measurement Health-related quality of life (HRQoL)

The EQ-5D is an instrument for measuring HRQoL. It is a simple scale developed by the EuroQoL Group for the measurement of overall health. It is currently being used in numerous countries after validation and computation of weighted values corresponding to the cultural values (20). It provides a single index score for the health state after measuring and adjusting these 5 health states, used to measure HRQoL (10). We used the model developed by the KCDC (21), which applied the weighted values for HRQoL in the Korean context, for calculating the EQ-5D index score. The EQ-5D index scores ranges from −1 to +1; −1, 0, and +1 indicating severe, moderate, and no problem respectively (19). The internal reliability of the scale (Cronbach’s ɑ) was .78 in a previous study (22), and .84 in this study.

### Physical environment

Physical environment was measured by the subjects’ positive attitudes toward their local community. It comprised of 5 items measured using a dichotomous scale (Satisfied-1 point, Unsatisfied-0 point): satisfaction with the “level of safety,” “natural environment,” “living environment,” “traffic condition,” and “access to health services.” The physical environment was measured by the sum of all the responses.

### General Characteristics

The general characteristics were examined in terms of demographic and health-related characteristics. For demographic characteristics, age, sex, educational level, marital status, employment status, and residential area were surveyed. For health-related characteristics, the subjective health status, experience of depression, degree of obesity, comorbidity, and experience of accidents were surveyed. Subjective health status measured on a 5-point Likert scale (5 for very healthy, 4 for healthy, 3 for average, 2 for unhealthy, and 1 for very unhealthy), with a higher score indicating a better subjective health status. Experience of depression was defined as having experienced depression in their daily life for more than two weeks in a year; measured as: ”not depressed” or “experienced depression.” Degree of obesity measured with the body mass index (BMI), calculated by dividing the body weight (kg) by the square of the height (m^2^). BMI<18.5 was classified as “underweight,” 18.5∼<23.0 as “normal weight,” 23.0∼<25.0 as “overweight,” and ≥25.0 as “obese” (23). Comorbidity was the number of chronic diseases diagnosed by a physician (i.e., hypertension, diabetes, arthritis), measured as “no disease,” “one disease,” “two diseases,” and “three diseases.” Experience of accidents was the number of accidents, addictions, and fall injuries sustained in the past year, measured as ”no accidents, addictions, and fall injuries,” “experienced once,” and “ experienced twice.”

### Statistical analysis

Data were analyzed using SPSS (Version 22, IBM, Armonk, NY). The general characteristics of the subjects were analyzed using frequency and percentage. The difference in the EQ-5D by general characteristics was identified using the t-test and analysis of variance. The correlations between the EQ-5D and related variables were analyzed through Pearson’s correlation. Hierarchical multiple regression analyses were used to predict the EQ-5D. Demographic characteristics (age, sex, educational level, marital status, employment status, residential area) were entered in Model I, and health-related variables (subjective health status, experience of depression, degree of obesity, comorbidity, experience of accidents) were entered in Model II. In addition, environmental variable (physical environment) was entered in Model III to identify the effects of the variables entered in each model. The general characateristics were coded as dummy variables, except for comorbidity and experience of accidents.

The Durbin-Watson statistic was 1.914, indicating no autocorrelation in the residuals, and correlation coefficients ranged from 0.047∼0.249, indicating independence. Tolerance ranged from 0.741∼0.980, and variance inflation factor (VIF) ranged from 1.021∼1.350, which indicates an absence of multicollinearity, satisfying the basic assumptions of regression.

## Results

### The EQ-5D scores according to the general characteristics

Demographic characteristics were significant differences in the degree to which the EQ-5D scores were distributed according to age, sex, educational level, marital status, employment status, and residential area (All *P*<0.001). Health-related variables showed significant differences in the degree to which the HRQoL varied in accordance with the subjective health status (*P*<0.001), experience of depression (*P*<0.001), degree of obesity (*P*<0.01), comorbidity (*P*<0.001), and experience of accidents (*P*<0.001). In addition, there were statistically significant differences in environmental variable, level of safety (*P*<0.01), natural environment (*P*<0.01), living environment (*P*<0.01), traffic condition (*P*<0.001), and access to health services (*P*<0.001) (Table 1).

**Table 1:** The EQ-5D scores according to the general characteristics of the subjects (n=3537)

***Characteristics***		***n(%)***	***M (SD)***	***t/ F(P)***
Age (years)	65–74	1687 (47.7)	0.73 (0.17)	−9.087 (<0.001)
	≥75	1850 (52.3)	0.68 (0.19)	
Sex	Male	1065 (30.1)	0.74 (0.20)	−8.437 (<0.001)
	Female	2472 (69.9)	0.69 (0.18)	
Educational level	≤Elementary school	1436 (40.6)	0.67 (0.18)	26.919 (<0.001)
	Middle school	1380 (39.0)	0.71 (.019)	
	High school	371(10.5)	0.73 (0.18)	
	≥College	350 (9.9)	0.76 (0.19)	
Marital status	Married	1260 (35.6)	0.71(0.20)	16.386 (<0.001)
	Widowed	1851 (52.3)	0.69 (0.17)	
	Divorced/separated/single	426 (12.1)	0.74 (0.18)	
Employment status	Employed	544 (15.4)	0.78 (0.14)	−11.309 (<0.001)
	Unemployed	2993 (84.6)	0.69 (0.19)	
Residential areas	Province	2574 (72.8)	0.70 (0.19)	−3.639 (<0.001)
	Metropolitan city	963 (27.2)	0.72 (0.17)	
Subjective health status	Very unhealthy	938 (26.5)	0.58 (0.21)	278.688 (<0.001)
	Unhealthy	1473 (41.7)	0.70 (0.13)	
	Average	772 (21.8)	0.81 (0.14)	
	Healthy	313 (8.8)	0.84 (0.14)	
	Very healthy	41 (1.2)	0.86 (0.13)	
Experience of depression	Have experienced	522(14.8)	0.62 (0.21)	−12.082 (<0.001)
	Not experienced	3015 (85.2)	0.72 (0.17)	
Degree of obesity †	Underweight	279 (10.3)	0.70 (0.19)	4.788 (0.002)
	Normal weight	1268 (46.8)	0.72 (0.18)	
	Overweight	589 (21.8)	0.73 (0.17)	
	Obese	572 (21.1)	0.70 (0.18)	
Comorbidity†	None	824 (23.3)	0.77 (0.18)	78.971 (<0.001)
	One disease	1371 (38.8)	0.72 (0.17)	
	Two diseases	1062 (30.1)	0.66 (0.19)	
	Three diseases	274 (7.8)	0.62 (0.18)	
Experience of Accidents†	None	2298 (65.1)	0.73 (0.18)	68.540 (<0.001)
	Once	936 (26.5)	0.67 (0.18)	
	Twice	298 (8.4)	0.62 (0.19)	
Level of safety	Satisfied	2901 (82.0)	0.70 (0.18)	3.471 (0.001)
	Unsatisfied	636 (18.0)	0.68 (0.19)	
Natural environment	Satisfied	3077 (87.0)	0.71 (0.18)	3.417 (0.001)
	Unsatisfied	460 (13.0)	0.68 (0.20)	
Living environment	Satisfied	2966 (83.9)	0.71 (0.18)	3.433 (0.001)
	Unsatisfied	571 (16.1)	0.68 (0.19)	
Traffic condition	Satisfied	2299 (65.0)	0.72 (0.18)	7.030 (<0.001)
	Unsatisfied	1238 (35.0)	0.67 (0.19)	
Access to health services	Satisfied	2443 (69.2)	0.71 (0.18)	4.720 (<0.001)
	Unsatisfied	1094 (30.8)	0.68 (0.20)	

Note: M: Mean, SD: Standard Deviation,

† missing data involved.

### Relations among major variables

The EQ-5D scores were significantly negatively correlated with comorbidity (*P*<0.001) and experience of accidents (*P*<0.001), and positively correlated with physical environment (*P*<0.001) (Table 2).

**Table 2:** Correlation between the EQ-5D, comorbidity, experience of accidents, and physical environment.

***Variable***	***EQ-5D***	***Comorbidity r (P)***	***Experience in accidents***	***Physical environment***
EQ-5D	1.00			
Comorbidity	−0.249(<0.001)	1.00		
Experience of accidents	−0.192 (<0.001)	0.091 (<0.001)	1.00	
Physical environment	0.119 (<0.001)	−0.008 (0.647)	−0.038 (0.023)	1.00

### Factors affecting the EQ-5D scores

First, among demographic characteristics, age (*P*<0.001), sex (*P*<0.001), educational level (*P*<0.01), marital status (*P*<0.05), employment status (*P*<0.001), and residential area (*P*<0.001) were found to have significant associations, with an explanatory power of 8.1% (*P*<0.001). Second, when health-related variables were entered in Model I, the demographic variables were significant and subjective health status (*P*<0.001), experience of depression (*P*<0.001), degree of obesity (*P*<0.01), comorbidity (*P*<0.001), and experience of accidents (*P*<0.001) were associated, with the explanatory power of Model II being 20.6% (*P*<0.001). When the environmental variable was entered in Model II, physical environment was significant (*P*<0.001), and the explanatory power of Model III was 21.4% (*P*<0.001) (Table 3).

**Table 3:** Effect of the variables on the EQ-5D score

***Predictors***		***Model I***		***Model II***		***Model III***	
***Variables***	***Categories (Reference group)***	***ß***	***t (P)***	***ß***	***t(P)***	***ß***	***t(P)***
Demographic factors	Age (≥75)	−.146	−8.730 (<0.001)	−.166	−10.628 (<0.001)	−.168	−10.818 (<0.001)
	Sex (male)	.121	6.719 (<0.001)	.054	3.120 (0.002)	.059	3.458 (0.001)
	Educational level (≥college)	.049	2.900 (0.004)	.032	2.038 (0.042)	.036	2.278 (0.023)
	Marital status (with spouse)	−.044	−2.497 (0.013)	−.038	−2.329 (0.020)	−.041	−2.504 (0.012)
	Employment status (employed)	.167	10.043 (<0.001)	.117	7.508 (<0.001)	.114	7.317 (<0.001)
	Residential area (metropolitan)	.058	3.499 (<0.001)	.053	3.432 (0.001)	.049	3.199 (0.001)
Health factors	Subjective health status (≥healthy)			.177	11.496 (<0.001)	.173	11.269 (<0.001)
	Experience of Depression (have)			−.149	−9.763 (<0.001)	−.141	−9.236 (<0.001)
	Degree of obesity (normal)			.041	2.680 (0.007)	.039	2.610 (0.009)
	Comorbidity			−.159	−10.069 (<0.001)	−.159	−10.135 (<0.001)
	Experience of Accidents			−.135	−8.859 (<0.001)	−.133	−8.759 (<0.001)
Environment factor	Physical environment					.092	6.134 (<0.001)
Const.(B)		.989	26.509 (<0.001)	1.097	30.975 (<0.001)	1.050	29.131 (<0.001)
F			53.0899 (<0.001)		84.292 (<0.001)		81.209 (<0.001)
R^2^			.083		.209		.217
Adjusted R^2^			.081		.206		.214
R^2^ change			.083		.126		.008

## Discussion

In terms of demographic characteristics, the EQ-5D score was higher among the younger elderly (65–74 yr), men, college graduates or higher, employed, living in a large city, and had no spouse. Our results were partially similar to previous studies (1, 9, 10, 19, 24, 25) that found significant effects on the HRQoL of the Korean low-income elderly population. With regard to marital status, precedent studies have shown that the presence of a spouse was significantly associated with higher HRQoL, which was the opposite of that in our study (9, 19, 26); however, in Spain (11), single individuals had higher QoL than married or widowed individuals did. Unlike previous studies, in our study, subjects who were voluntarily single (divorced, separated, unmarried) had a significantly better QoL. Additional replication studies and meta-analysis would be required to elucidate whether this result is a group-specific phenomenon that is only demonstrated among low-income elderly, or if it is an accurate reflection of the rising “twilight divorce” and unmarried rates in the Korean society. An antecedent study recommends conducting more studies to investigate the influence of marriage on HRQoL (27).

Among the health-related variables, the EQ-5D score was higher among individuals with better subjective health status, no experience of depression, normal body weight, less comorbidity, and no experience of accidents. Subjective health status was found to be the most significant predictor of HRQoL among the Korean NBLSS beneficiaries. It was also found to be a significant influence factor of HRQoL in previous studies on disadvantaged citizens in rural areas (5), female elderly NBLSS recipients who live alone (3), and frail elderly citizens (28). As respondents with low socioeconomic status have low expectations for health, which affects the EQ-5D score, it is important to devise policies that minimize adverse health outcomes caused by socioeconomic inequality (10).

Comorbidity was found to be a significant predictor of HRQoL. This was similar to the results suggested by precedent researches, where the EQ-5D score was lower among individuals with comorbidity (10, 12, 24, 25, 29). As a measure of comorbidity in this study, we examined the prevalence of hypertension, diabetes, and arthritis-3 diseases that are generally highly prevalent and are under national management (12). In addition to the effects in the health-related aspects, management of these diseases also contributes to the improvement of QoL among low-income elderly persons, necessitating more active chronic disease management. In our study, comorbidity was presented as the number of diseases present, which does not reflect the severity of the diseases (26); thus, future studies should consider using objective indicators of comorbidity, such as the Cumulative Illness Rating Scale or Charlson Comorbidity Index (24).

Depression has a greater effect on the QoL than health problems or behaviors among the low-income elderly population (1, 10), suggesting that physical or psychological factors threaten QoL in the low-income elderly population than in the general elderly population. With regard to degree of obesity, the normal group had a better EQ-5D score than that of the abnormal group. This was similar to a previous study on the HRQoL of elderly citizens who regularly visited welfare centers (19), controlling abnormal obesity degree among the elderly is important for managing their HRQoL, and subjects classified into normal and abnormal groups instead of merely examining them based on the BMI values. The absence of experiences of accidents was associated with a higher EQ-5D score. Factors related to independence are prioritized with regard to the HRQoL of the elderly population (30), accidents that affect independence are speculated to have adverse effects on the HRQoL. Particularly, fall injuries restrict the mobility of elderly persons and ultimately undermine their HRQoL (8, 30), supporting the findings of this study.

With regard to environmental variable, physical environment was found to be a significant predictor of the HRQoL in the elderly NBLSS beneficiaries. Individuals who reside in physically inadequate environments perceive their environment negatively and ultimately show low HRQoL. The satisfaction with the area of residence and neighborhood environment has been confirmed to affect life satisfaction of the elderly (31). Subjective health status was higher in groups satisfied with their physical environment, suggesting that improving the community environments would bring about better health outcomes (15, 17). Therefore, continued interest is important to encourage local governments to implement community environmental improvement projects and help local residents develop positive perceptions about their environment (32). We measured the presence of satifsfaction about the level of safety, natural environment, living environment, traffic condition, and access to health services, but future research should be apply the appropriate assessment tools to the physical environment. Specifically, the management of HRQoL in terms of the physical environment requires a multidisciplinary approach. Experts in the field related to the physical environment of local residents are required to enhance the understanding of HRQoL. Further, undergraduate programs should start a course on health and QoL, and basic education should be provided integrating this. In addition, continuous exchange of information among specialized disciplines, such as organizing multidisciplinary academic conferences, are necessary.

This study was a secondary analysis of a cross-sectional survey and there was a limit to the predictors and use of appropriate instruments in the prior study. Therefore, this study will need to be repeated using appropriate tools to target populations. Despite this limitation, this study provided meaningful contributions by identifying the demographic factors, health-related factors, and physical environment that have significant effects on the HRQoL of elderly NBLSS beneficiaries.

## Conclusion

To improve the HRQoL of elderly NBLSS, multilateral efforts that involve considerations of demographic and health-related variables at the individual level and associations with the physical environment at the community level are required. In the community, we must seek out various ways to improve the elements that constitute the physical environment. Further, the management of subjective health status was important to improve the HRQoL of the elderly NBLSS beneficiaries. For the vulnerable NBLSS beneficiaries, systematic approaches to regional health policy and healthier community environments are required.

## Ethical considerations

Ethical issues (Including plagiarism, informed consent, misconduct, data fabrication and/or falsification, double publication and/or submission, redundancy, etc.) have been completely observed by the authors.
